# Automatic Recognition and Prognostic Prediction of Colorectal Liver Metastases Using a Multi-Scale Deep Learning Framework: Model Development and Validation Study

**DOI:** 10.2196/73311

**Published:** 2026-04-07

**Authors:** Houwen Long, Feng Ying, Shi Wu, Long Li

**Affiliations:** 1 Department of Anus and Intestine Surgery, Yongkang First People’s Hospital Affiliated to Hangzhou Medical College, 599 Jinshan West Road, Dongcheng Street, Yongkang, 321300, China, 86 0579-89279021

**Keywords:** colorectal liver metastasis, deep learning, pathological images, prognostic prediction, model evaluation, integrated learning

## Abstract

**Background:**

Colorectal cancer liver metastasis (CRLM) presents considerable challenges in both diagnosis and prognosis, as conventional approaches often are limited by subjectivity, variability, and limited efficiency. Recent advances in deep learning have shown great potential for automated extraction of pathological features, offering improved diagnostic accuracy and more reliable prognostic predictions.

**Objective:**

This study aimed to develop and validate a multi-model ensemble deep learning framework (colon cancer liver metastasis network [CLM-Net]) for the automatic recognition and prognostic prediction of CRLM from pathological images, thereby enhancing diagnostic accuracy and clinical applicability.

**Methods:**

A total of 197 pathologically annotated CRLM cases were collected and integrated from publicly available datasets (Kaggle and The Cancer Imaging Archive) to construct high-quality training and independent test sets. CLM-Net was built upon base convolutional neural network architectures including VGG16, DeepLab-v3, and U-Net, incorporating multi-scale atrous convolutions, a squeeze-and-excitation attention mechanism, a conditional random field refinement module, and transfer learning strategies. The framework was comprehensively evaluated using 5-fold cross-validation and independent testing for classification, segmentation, and prognostic prediction tasks. For survival prediction, 1024-dimensional image feature vectors extracted by CLM-Net were analyzed using logistic regression and random forest classifiers, with Kaplan-Meier curves and log-rank tests used for survival analysis.

**Results:**

CLM-Net demonstrated superior performance in pathological image recognition, achieving 94% accuracy, 92% recall, 93% *F*_1_-score, and an area under the receiver operating characteristic curve of 0.96 on the independent test set, outperforming single models. For survival prediction, CLM-Net with multi-scale attention achieved an area under the receiver operating characteristic curve of 0.864. Kaplan-Meier analysis revealed its significantly stronger risk stratification ability compared with VGG16, U-Net, and DeepLab-v3. Precision-recall curves and heatmaps further confirmed the model’s high robustness and generalizability in unseen samples. Clinical evaluation by pathologists indicated strong interpretability and diagnostic utility, with a concordance rate of 90%.

**Conclusions:**

The proposed CLM-Net framework, through the integration of diverse deep learning architectures and mechanisms, markedly improved the recognition and prognostic prediction of CRLM, demonstrating excellent generalization and clinical translation potential. Future studies will focus on multi-center validation and integration of multimodal features to further optimize its role in precision medicine.

## Introduction

Colorectal cancer (CRC) is among the most prevalent malignant tumors worldwide, with liver metastasis being a major cause of mortality in affected patients [1-3]. In high-income countries in particular, both the incidence and mortality rates of CRC remain high. According to global cancer statistics, CRC consistently ranks among the top cancers in terms of incidence, and approximately 25% to 50% of patients with CRC will eventually develop liver metastases. Hepatic metastasis not only represents the primary driver of CRC-related death but also markedly affects patient quality of life and treatment efficacy. Early and accurate diagnosis, as well as reliable prediction of colorectal cancer liver metastasis (CRLM), is therefore essential for improving patient outcomes and guiding personalized therapeutic strategies [4-6]. Deep learning, a leading technology in the field of artificial intelligence, has demonstrated great potential in this area. By leveraging large-scale pathological image datasets, deep learning algorithms can autonomously learn and extract clinically relevant features, thereby enhancing the diagnostic precision and prognostic assessment of CRLM [7,8].

In recent years, the rapid advancement of deep learning technologies has revolutionized medical image analysis [9,10], enabling the efficient interpretation of complex pathological data through automated feature extraction [11]. Despite these advances, substantial gaps remain in the application of deep learning to the diagnosis and prognostic prediction of CRLM. Most existing studies rely on single deep learning models, which often struggle to accurately capture complex pathological features such as tumor boundaries and microarchitectural details. This limitation compromises the precision and robustness of predictive outcomes. Furthermore, how to effectively integrate the strengths of different deep learning algorithms to construct more comprehensive and reliable models remains an urgent challenge [8,12].

In recent years, deep learning techniques have been extensively applied to the diagnosis and prognosis prediction of CRLM. Most existing studies have relied on single-model frameworks—such as U-Net, ResNet, or temporal long short-term memory networks—for lesion segmentation, image classification, or dynamic disease prediction, respectively. However, these approaches often experience limited feature extraction capabilities, an inability to simultaneously address both diagnostic and prognostic tasks, and poor adaptability to the inherent heterogeneity of pathological images. To provide a comprehensive overview of the current landscape, we systematically reviewed representative deep learning-based studies in CRLM analysis, summarizing their specific tasks, data sources, advantages, and limitations (Table S1 in Multimedia Appendix 1). In contrast, the colon cancer liver metastasis network (CLM-Net) model proposed in this study integrates the architectural strengths of VGG16, DeepLab-v3, and U-Net, and incorporates multi-scale feature fusion, squeeze-and-excitation (SE) attention modules, and a conditional random field postprocessing component. These enhancements significantly improve image recognition performance while enabling accurate prognostic prediction and robust clinical applicability, effectively addressing the limitations of existing single-task models.

To overcome these challenges, we designed and developed a novel multi-model ensemble deep learning framework, CLM-Net, designed to enhance both the efficiency and accuracy of pathological image analysis in CRLM. CLM-Net harnesses the strengths of multiple deep learning architectures, including convolutional neural networks (CNNs) (eg, VGG16), segmentation models (eg, DeepLab-v3), and multi-scale processing modules. The model integrates multi-resolution pathological features to enhance representational power and generalizability. In the feature extraction stage, an SE attention mechanism is introduced to strengthen the model’s focus on key lesion areas, thereby improving analysis precision. Additionally, the adoption of transfer learning not only accelerates model convergence but also enhances performance on limited-sample datasets.

CLM-Net is designed to address critical challenges in the diagnosis and prognostic evaluation of CRLM by automatically extracting and integrating microscopic features from pathological images, in combination with relevant clinical indicators. This integrated approach provides a highly efficient and accurate prediction tool. The primary objective of this study is to enhance diagnostic precision and prognostic assessment for CRLM, while overcoming limitations of current models in feature representation and overall performance.

This work not only bridges a significant technological gap in the field of CRLM diagnosis and prognosis but also opens new avenues for the development of personalized medicine. By coupling pathological feature extraction with predictive analytics, CLM-Net holds strong potential as a valuable tool in precision oncology for CRLM, contributing to both technological innovation and improved clinical decision-making.

In summary, we propose CLM-Net, a novel deep learning framework that incorporates multi-model ensemble learning, attention mechanisms, and transfer learning to enhance automated recognition and prognostic prediction of CRLM pathology images. We hypothesize that CLM-Net will demonstrate superior performance in diagnostic accuracy, prognostic capability, and generalizability compared with conventional single-model approaches and will be highly endorsed by clinical professionals for its practical utility.

## Methods

### Ethical Considerations

This study was conducted using publicly available datasets, including the Liver Tumor Segmentation dataset from Kaggle and The Cancer Imaging Archive (TCIA). Because the data are publicly accessible and fully deidentified, institutional review board approval was not required for this retrospective study in accordance with institutional policies of Yongkang First People’s Hospital Affiliated to Hangzhou Medical College (policy document available upon request). The study was conducted in accordance with the ethical principles outlined in the Declaration of Helsinki.

All datasets used in this study contain anonymized patient information, and no identifiable personal data were accessed. Therefore, informed consent from individual participants was not required.

The study adhered to principles of data privacy and confidentiality, as only deidentified imaging and clinical metadata were used for research purposes.

No financial or other compensation was provided to participants, as this study did not involve direct patient recruitment or intervention.

### Public Data Download and Integration

The pathological image data used in this study were primarily derived from 2 public databases: the Liver Tumor Segmentation Challenge dataset on Kaggle and TCIA. The Kaggle dataset contained 129 CRLM-related histopathological slides with corresponding clinical information, including age, sex, and tumor stage. To further enhance the sample size and diversity, an additional 68 CRLM cases were obtained from TCIA. In total, image data from 197 patients were included. After quality control and image augmentation, a final set of 1000 high-quality images was generated for model training and evaluation.

During preprocessing, low-resolution or unevenly stained images were excluded. All images were resized to 256×256 pixels to meet the input requirements of the model. Annotations were performed by experienced pathologists to ensure the accuracy of metastatic lesions and surrounding regions. Each image was linked to a unique patient identifier to guarantee patient-level independence across training, validation, testing, and prognostic modeling tasks.

### Detailed Annotation of Pathological Images

Pathological image annotation was conducted by board-certified pathologists with over 10 years of professional experience. Using Aperio ImageScope software (version 12.3.3; Leica Biosystems, Germany), the experts meticulously annotated tumor regions and liver metastasis features within each slide. In addition, they recorded relevant clinical parameters, including tumor size and morphology, clarity of tumor boundaries with adjacent normal liver tissue, the spatial distribution of tumor-associated macrophages, and the density and localization of immune cells (eg, T cells and B cells) within the tumor microenvironment. All annotation data were saved in XML format to ensure compatibility and efficient parsing by the subsequent deep learning models.

### Independent Verification and Annotation Error Control

To ensure annotation accuracy, all pathological images in this study were independently annotated by 2 experienced pathologists. Interrater reliability was assessed using the Cohen κ coefficient. In cases of disagreement, a third senior pathologist reviewed and confirmed the final annotation, ensuring both precision and consistency. To minimize manual annotation errors, the following quality control strategies were implemented:

- Dual annotation and cross-validation: Each image was independently annotated by 2 pathologists, and results were cross-verified to reduce single-observer bias.
- Standardized training protocols: All participating pathologists underwent unified training and followed standardized annotation guidelines to ensure interobserver consistency.
- Tool-assisted annotation: Semiautomated annotation tools were used to assist in lesion identification, thereby enhancing both efficiency and accuracy.

These measures collectively ensured high-quality annotations, establishing a robust and reliable dataset for subsequent deep learning model training and analysis.

### Application of Data Augmentation Techniques

To improve model generalization across diverse pathological image features, various data augmentation strategies were applied, tailored to the specific requirements of each deep learning architecture. For VGG16, images were first resized to 224×224 pixels using bilinear interpolation to match the model’s input specification. Standard normalization was then performed by subtracting the dataset’s mean pixel value and dividing by the SD, thereby transforming the pixel distribution to approximate a standard normal distribution. In the case of DeepLab-v3, numerical data were similarly normalized to a mean of 0 and an SD of 1. For textual data inputs, word embedding techniques were used to convert words into dense vector representations using pretrained models such as Word2Vec or GloVe for initialization. For U-Net, input images were also resized and normalized as described above. In addition, random cropping and horizontal flipping were implemented to increase data variability and reduce overfitting. All preprocessing steps were conducted using standard APIs provided by mainstream deep learning frameworks such as TensorFlow and PyTorch. Specifically, the torchvision.transforms module in PyTorch was used for image transformations.

### Development of Basic Convolutional Neural Network Models

Several classical CNN models, including DeepLab-v3, VGG16, and U-Net, were used for the classification and segmentation of pathological images. All models were implemented using the PyTorch framework (version 1.7.1; Meta AI, Meta Platforms, Inc). To accelerate convergence and improve generalization, model parameters were initialized with pretrained weights from the ImageNet dataset. Training was conducted using the Adam optimizer, with a learning rate of 1×10^–4^, a batch size of 32, and 50 training epochs.

### DeepLab-V3

**Figure 1.**
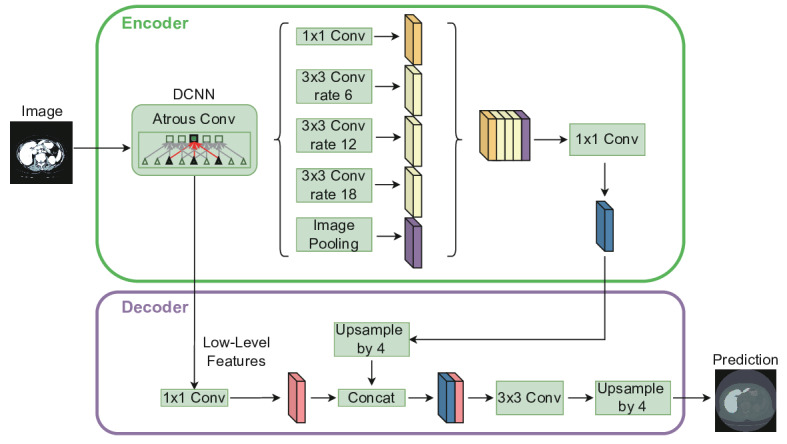
Schematic diagram of the DeepLab-v3 algorithm. DCNN: deep convolutional neural network.

DeepLab-v3 is a state-of-the-art semantic segmentation model that incorporates dilated (atrous) convolutions to capture multi-scale contextual information without reducing spatial resolution. Its core component, atrous spatial pyramid pooling (ASPP), applies parallel atrous convolutions with varying dilation rates to extract rich semantic features at multiple scales, thereby enhancing model adaptability to complex structures in histopathological images. The use of dilated convolutions expands the receptive field without increasing the number of parameters or computational cost, enabling more comprehensive feature extraction. DeepLab-v3 follows an encoder-decoder architecture, where the encoder captures high-level semantic features, and the decoder restores spatial details for precise segmentation. Commonly, pretrained backbone networks such as Xception or MobileNet are adopted to serve as feature extractors, followed by the ASPP module and decoder to complete the segmentation task. The model structure is illustrated in Figure 1.

### VGG16

**Figure 2.**
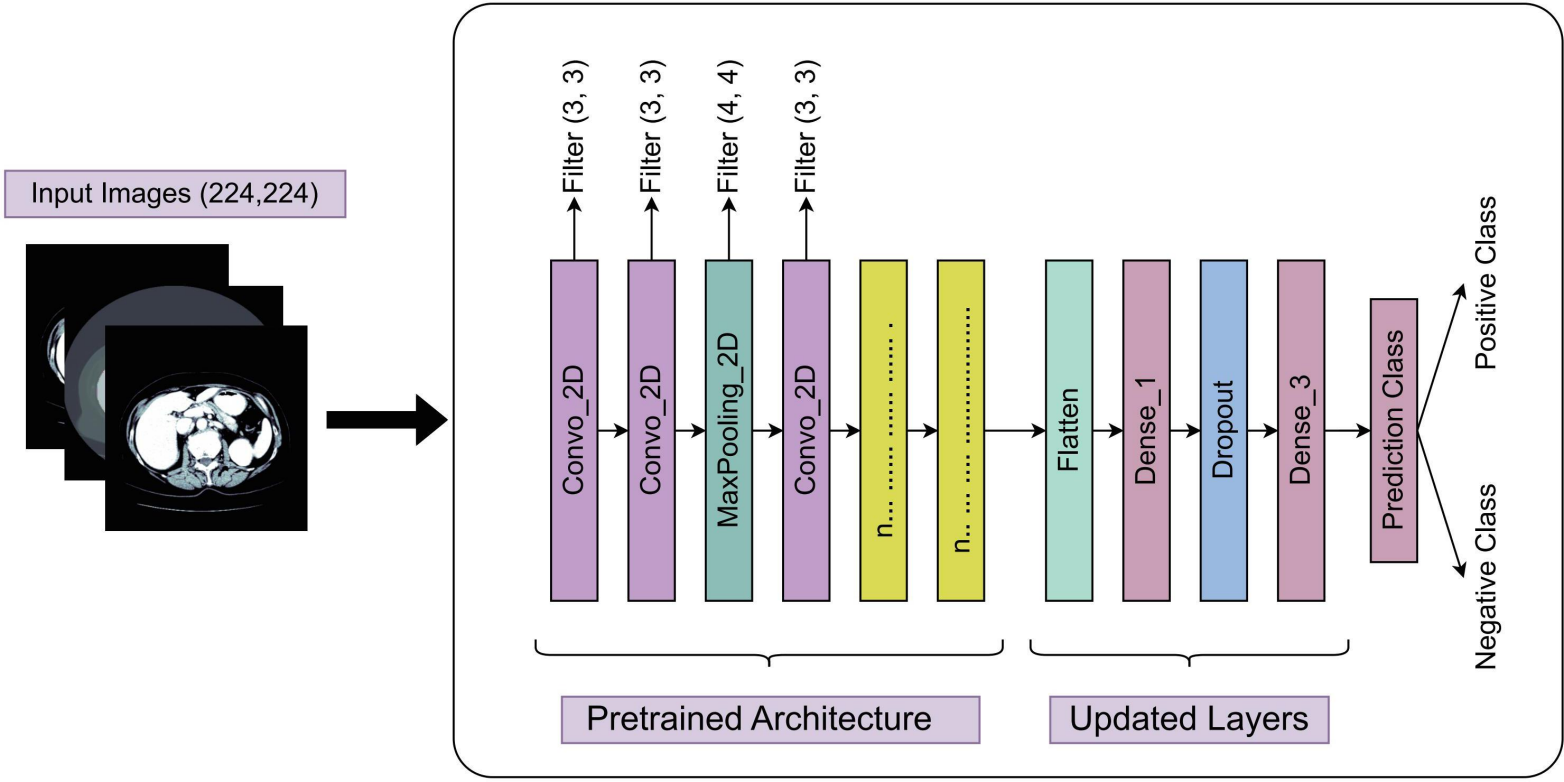
Schematic diagram of the VGG16 algorithm.

VGG16 is a classic deep CNN composed of 16 layers, including 13 convolutional layers and 3 fully connected layers. It captures image features by repetitively stacking 3×3 convolutional kernels and 2×2 max-pooling layers. By using multiple small kernels, VGG16 achieves a larger receptive field while maintaining a relatively low number of parameters and a deeper network structure. Its architecture is straightforward and consistent, with fixed strides in all convolutional and pooling layers, which simplifies network design and optimization. In our study, VGG16 was initialized with pretrained weights from the ImageNet dataset. During training, images were preprocessed by mean subtraction and optimized using mini-batch stochastic gradient descent. The algorithmic framework is illustrated in Figure 2.

### U-Net

**Figure 3.**
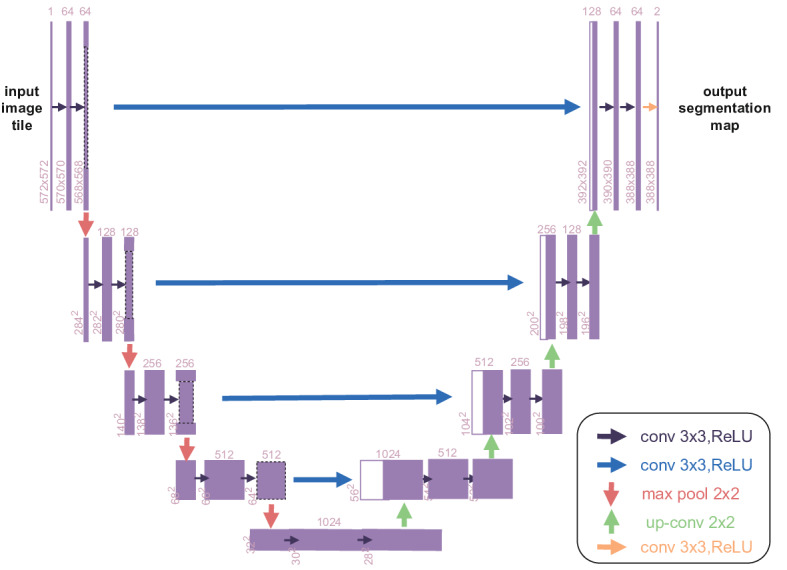
Schematic diagram of the U-Net algorithm. ReLU: rectified linear unit.

U-Net is a neural network model specifically designed for medical image segmentation, characterized by its U-shaped architecture consisting of a contracting path (encoder) and an expansive path (decoder). A key feature of U-Net is the use of skip connections that merge encoder feature maps with corresponding decoder outputs, thereby preserving spatial context and enhancing the reconstruction of fine-grained details. U-Net relies heavily on data augmentation techniques during training, such as rotation, scaling, and elastic deformation, to improve generalizability. In our implementation, the model was trained using the Adam optimizer, with a learning rate of 1×10^–4^, a batch size of 8, and a cross-entropy loss function. The algorithm structure is shown in Figure 3.

Each of the 3 models demonstrates unique strengths for different image analysis tasks. DeepLab-v3 is suited for fine-grained segmentation and multi-scale feature extraction. VGG16, with its efficient and compact design, is often used as a backbone for various visual recognition tasks. U-Net, in contrast, excels in medical image segmentation, particularly in scenarios with limited datasets. Based on the complementary strengths of these architectures, we developed a customized CNN framework, CLM-Net, specifically tailored for the automatic recognition of pathological features in CRLM.

### Design of the CLM-Net Model

CLM-Net is a CNN framework specifically developed for the automatic recognition of pathological features in CRLM histopathological images. The architecture adopts a parallel fusion structure that integrates the strengths of 3 widely used CNN models: VGG16, DeepLab-v3, and U-Net. Initially, input images are processed through a VGG16 backbone pretrained on ImageNet to extract low-level texture features. These features are then passed into 2 parallel branches: a DeepLab-v3 path and a U-Net path. The DeepLab-v3 branch applies atrous convolutions with dilation rates of {1, 2, 4, 8}, combined with an ASPP module, to capture rich multi-scale contextual information. Meanwhile, the U-Net branch adopts an encoder–decoder structure with skip connections to preserve spatial resolution and boundary detail. Mid-level feature fusion is performed by channel-wise concatenation of outputs from the VGG16, DeepLab-v3, and U-Net bottleneck layers.

To further enhance feature discrimination, a SE attention module (reduction ratio *r*=16) is inserted after the fusion stage to adaptively recalibrate channel-wise feature importance, improving the model’s sensitivity to tumor-relevant features while suppressing background noise. The fused features are then passed through a unified decoder, and the final segmentation map is refined using a conditional random field module to enhance pixel-level consistency, particularly at lesion boundaries. In addition to the segmentation output, the decoder also generates a 1024-dimensional feature vector, which is used in downstream survival prediction tasks.

**Figure 4.**
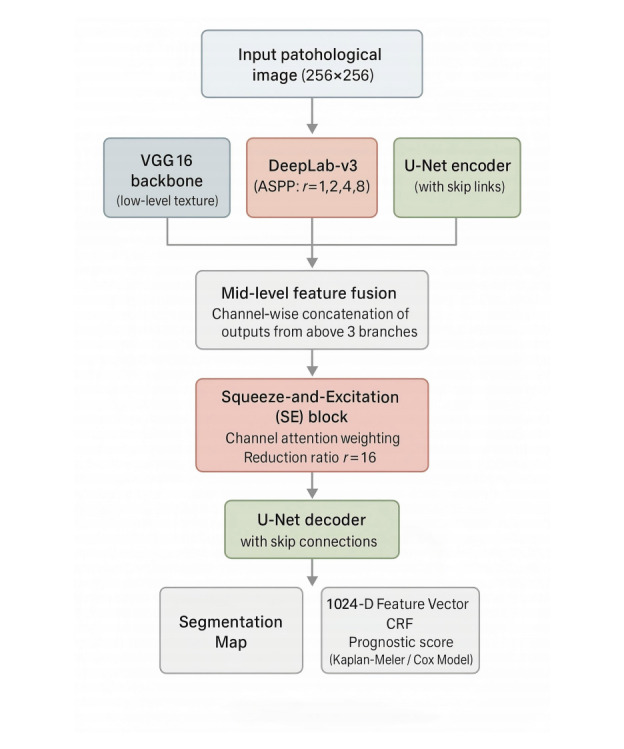
Schematic diagram of colon cancer liver metastasis network (CLM-Net) feature extraction. CLM-Net comprises 3 parallel encoding branches: VGG16 for low-level texture feature extraction; DeepLab-v3 with a multi-scale atrous convolution ASPP module (dilation rates=1, 2, 4, 8) for contextual feature extraction; U-Net encoder with skip connections to retain spatial details. The mid-level features from the 3 submodels are integrated via channel-wise concatenation, followed by an SE attention module (reduction ratio *r*=16) to enhance channel-specific weighting. The fused features are fed into a shared decoder, which leverages U-Net skip connections to further recover spatial information. The decoder generates 2 outputs: a segmentation map for pathological image analysis and a 1024-dimensional feature vector for survival prediction. The outputs are refined by the conditional random field module, with the feature vector subsequently used in Kaplan-Meier or Cox proportional hazards models to predict survival probabilities in colorectal cancer liver metastasis patients. ASPP: atrous spatial pyramid pooling; CRF: conditional random field; SE: squeeze-and-excitation.

During training, CLM-Net used the same hyperparameters as the base models, including the Adam optimizer, a learning rate of 1×10^–4^, a batch size of 32, and 50 training epochs. To address the limited size of the training dataset and improve model generalizability, extensive data augmentation techniques such as rotation, scaling, and elastic deformation were applied. By integrating low-level feature extraction (VGG16), multi-scale semantic context (DeepLab-v3), and spatial detail preservation (U-Net), CLM-Net provides a robust and comprehensive solution for accurate CRLM lesion segmentation and prognosis prediction. The overall framework is illustrated in Figure 4.

### Performance Evaluation Metrics Calculation

To comprehensively assess the performance of various deep learning models for CRLM pathological image recognition, several metrics were used, including accuracy, recall, precision, *F*_1_-score, and the area under the receiver operating characteristic curve (AUC). For prognostic analysis, survival-related assessments such as Kaplan-Meier curves were used. Parameter tuning was performed using a 5-fold cross-validation strategy. All metric computations were implemented in Python using the classification_report and roc_auc_score functions from the Scikit-learn library. Each model’s performance on an independent test set was carefully documented to ensure objective and reliable evaluation.

### Implementation of K-Fold Cross-Validation

To evaluate the generalization capability of each model, a k-fold cross-validation approach was adopted, with k set to 5. Specifically, the dataset was randomly partitioned into 5 equal subsets. In each iteration, one subset served as the validation set, while the remaining 4 were used for training. This process was repeated 5 times to ensure each subset was used once for validation. The cross-validation procedure was executed using the KFold class from the Scikit-learn library. This approach enabled a fair and comprehensive assessment of each model’s generalization performance.

### Model Performance Comparison and Optimal Model Selection

Upon completing training, validation, and evaluation, the models were compared based on average performance across all metrics, including accuracy, recall, *F*_1_-score, and AUC. Custom Python scripts were used to facilitate a standardized and transparent comparison process. The model demonstrating the most favorable overall performance was selected as the optimal model. Subsequent to selection, this model underwent further refinement, including hyperparameter tuning and expanded data augmentation strategies, to enhance prediction accuracy and clinical applicability.

### Training and Evaluation

All models were trained using the PyTorch 1.7.1 framework, with the Adam optimizer set at a learning rate of 1e−4, a batch size of 32, and for 50 training epochs to ensure adequate learning of data features. A transfer learning strategy was used by initializing model weights with pretrained parameters from the ImageNet dataset, which helped accelerate convergence and improve training stability. To comprehensively assess model performance, several evaluation metrics were used, including accuracy, recall, *F*_1_-score, and the area under the receiver operating characteristic (ROC) curve (AUC). For prognostic assessment, Kaplan-Meier survival curves were generated to evaluate the model’s ability to predict patient outcomes. All models were evaluated using 5-fold cross-validation. In this approach, the dataset was randomly divided into 5 equal subsets; during each iteration, one subset was designated as the validation set, and the remaining 4 served as the training set. This method ensured robust performance evaluation and validated the generalization capability of the models across different data partitions.

### Evaluation of an Independent Test Set

To ensure the generalizability of the model and the reliability of the evaluation results, the dataset was obtained from publicly available databases and randomly divided into training, validation, and independent test sets in a 70%‐15%‐15% ratio. The training set was used for model training, the validation set for hyperparameter tuning, and the independent test set for final performance evaluation. The training set included approximately 700 images, the validation set 150 images, and the independent test set 150 images, all of which were annotated by experienced pathologists. A balanced class distribution between liver metastasis (Class 1) and nonmetastasis (Class 0) was maintained across all subsets to ensure consistent class representation during training and fair performance evaluation. Detailed class distribution across subsets is provided in Table S2 in Multimedia Appendix 1. All images were sourced from publicly available CRLM pathology databases, including TCIA and Kaggle. Image quality and annotation accuracy were verified through multiple rounds of manual verification by expert pathologists.

On the independent test set, the CLM-Net model demonstrated outstanding performance. Compared with other baseline CNN models, CLM-Net achieved notably higher scores in accuracy, recall, and *F*_1_-score, indicating stronger generalization and robustness in recognizing pathological features of CRLM.

### Definition and Calculation of Matching Rate

In this study, the matching rate was defined as the overlap between the model’s predicted positive regions and the ground-truth annotated positive regions, calculated using the intersection over union (IoU). The specific formula is as follows: $IoU=\frac{Predicted\;Region\cup Ground\;Truth\;Region}{Predicted\;Region\cap Ground\;Truth\;Region}$.

To evaluate model performance, the IoU value for each pathological image in the independent test set was calculated. The overall matching rate was then evaluated by calculating the average IoU across the entire test set. The experimental results showed that the matching rate for all selected models exceeded 85%, with the CLM-Net model achieving the highest matching rate (close to 90%). This demonstrates the model’s strong regional localization ability. Moreover, the high consistency of the matching rate across different samples reinforces the reliability of CLM-Net in pathological image analysis tasks.

### Survey Design and Implementation

To assess the practical clinical applicability of the model, a standardized survey was designed and conducted. The survey comprised the following components: (1) quantitative assessment of the model’s prediction accuracy, (2) evaluation of usability and user experience through a structured questionnaire, (3) subjective feedback from clinicians regarding the model’s diagnostic assistance capabilities (via open-ended questions), and (4) suggestions for further improvement and optimization of the model (also via open-ended questions). The survey was administered to a cohort of 10 experienced pathologists and clinicians. Questionnaires were distributed electronically and collected during the model validation phase. The responses were analyzed using both quantitative statistical methods and qualitative text analysis, providing critical insights to guide subsequent refinements in model development and optimization.

### Evaluation of Prognostic Prediction Capability

To assess the prognostic prediction performance of the CLM-Net model in patients with CRLM, both traditional survival analysis methods and machine learning-based classifiers were used. Specifically, Kaplan-Meier survival curves and the Cox proportional hazards model were used to analyze survival outcomes across different patient groups. For prognosis prediction, the 1024-dimensional feature vectors were extracted from the final fusion layer of the CLM-Net encoder. These vectors integrated multi-scale representations from VGG16, DeepLab-v3 (with an ASPP module), and U-Net (with skip connections), further enhanced by an SE attention mechanism. To reduce dimensionality and suppress noise, 2 preprocessing strategies were adopted:

- Principal Component Analysis: Retained principal components explaining >95% of cumulative variance, typically preserving 80‐100 dimensions.
- Variance-based Feature Selection: Removed low-variance features using a threshold of 0.01.

The processed feature vectors were input into 2 classical machine learning models:

- Logistic Regression: A linear model used to evaluate the influence of specific features on survival risk, using L2 regularization. The regularization parameter (C) was optimized through cross-validation over values of 0.1, 1, and 10.
- Random Forest Classifier: A nonlinear ensemble model used to capture complex feature interactions. Model tuning was performed via grid search, varying the number of trees (100 to 500) and maximum depth (5, 10, and 20). The optimal configuration was determined to be 100 trees with a maximum depth of 10. Patients were stratified into 2 prognostic groups: short-term survival (<30 d) and long-term survival (≥30 d) groups. Classifier performance was evaluated using accuracy, AUC, Kaplan-Meier survival curves, with statistical significance assessed via log-rank tests.

For comparative evaluation, the same prediction framework was applied to features extracted from 3 baseline deep learning models—VGG16, DeepLab-v3, and U-Net. Each model generated survival prediction probabilities from the input pathological images. A threshold of 0.5 was used to classify samples into short- and long-term survival groups. Kaplan-Meier curves were plotted for each model using the lifelines Python library, with time on the x-axis and cumulative survival probability on the y-axis. The Log-rank test was used to determine statistically significant differences among survival curves, and the final comparative results are presented in Table S3 in Multimedia Appendix 1.

### Clinical Validation

This study was conducted as a retrospective analysis using publicly available datasets and did not involve any direct clinical trials or the use of patient data beyond those datasets. To assess the practical applicability of the model, we collaborated with clinicians from affiliated hospitals to evaluate its usability and diagnostic accuracy. Clinical experts reviewed the model’s pathological image analysis outputs and compared them with conventional histopathological interpretations to assess its potential value in diagnostic support. Feedback from these professionals was systematically collected, focusing on diagnostic consistency, ease of interpretation, and clinical relevance. Based on this expert feedback, iterative modifications and optimizations were applied to enhance the model’s clinical adaptability and reliability.

## Results

### Superior Performance of the CLM-Net Ensemble Model in CRLM Pathological Image Recognition

**Figure 5.**
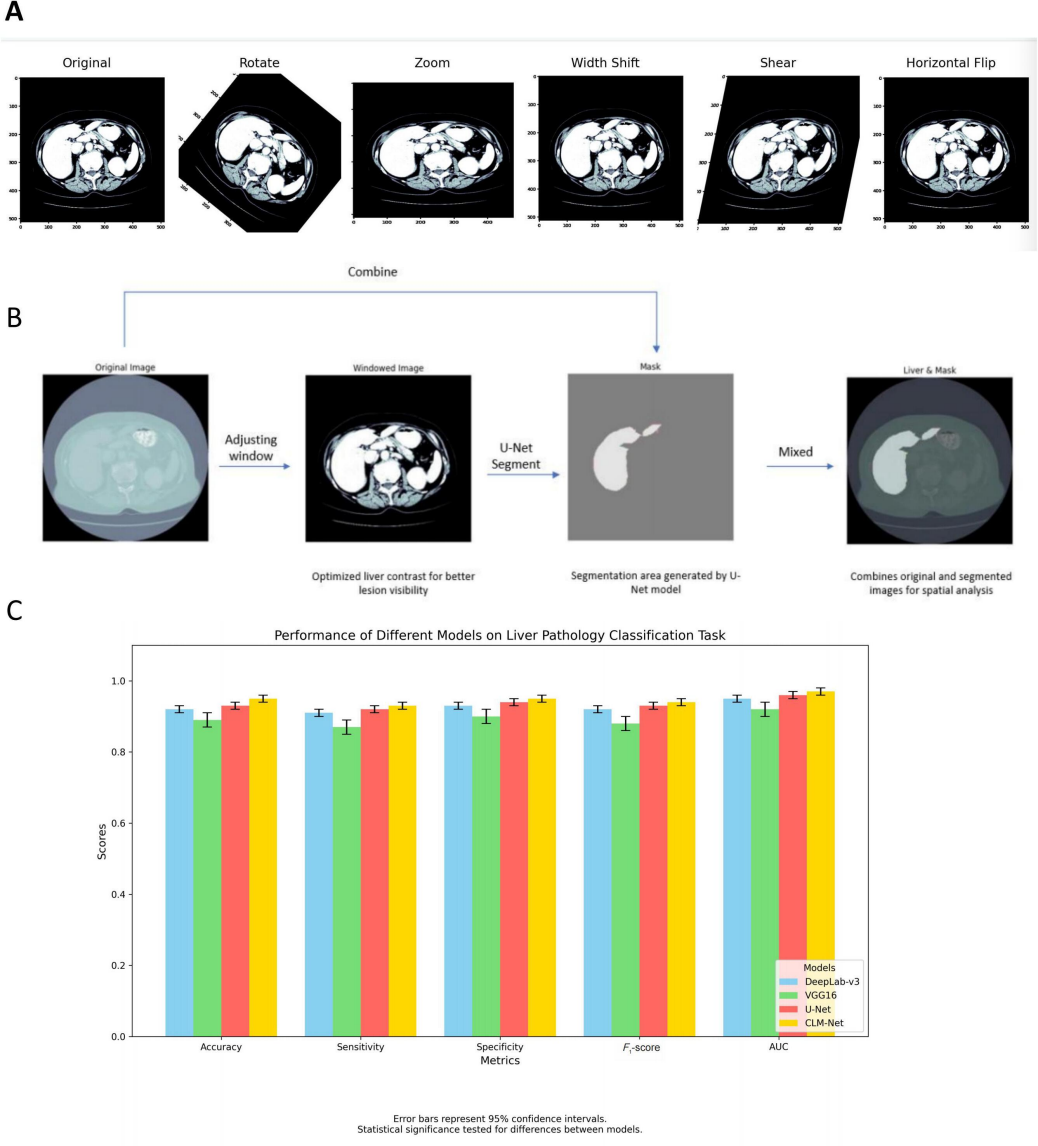
Comparative analysis of the CLM-Net model recognition process and experimental results on colorectal cancer liver metastasis (CRLM) pathological images. (A) Data augmentation techniques, such as random rotation, flipping, and cropping, enhance model training by increasing image diversity, improving generalization, and robustness in detecting complex patterns in CRLM images. (B) The recognition process involves preprocessing, feature extraction, and model integration, which enhances diagnostic accuracy and reliability in CRLM image analysis, providing valuable clinical insights. (C) A comparison of CLM-Net and individual models (VGG16, DeepLab-v3, and U-Net) shows that CLM-Net outperforms them in key metrics (accuracy, recall, *F*_1_-score, and AUC), owing to its ability to synthesize different model strengths, enhanced by data augmentation. Error bars represent 95% CIs. Statistical significance was tested for differences between models. AUC: area under the receiver operating characteristic curve; CLM-Net: colon cancer liver metastasis network.

Throughout the model development and validation process, data augmentation strategies significantly enhanced the performance of all deep learning models, particularly the ensemble learning model. The application of random rotations, horizontal flips, and cropping effectively increased training data variability, thereby improving model robustness and generalizability to previously unseen pathological images (Figure 5A). This approach is especially critical in the context of CRLM, where metastatic lesions are known to be highly heterogeneous in appearance.

The ensemble learning model (CLM-Net) outperformed individual models across multiple evaluation metrics by leveraging the complementary strengths of each architecture. While certain base models, such as U-Net, excelled at delineating fine-grained tumor boundaries, others (eg, DeepLab-v3) were more proficient in identifying contextual and structural features of metastatic lesions. By integrating these capabilities, CLM-Net achieved a more comprehensive and accurate recognition of CRLM pathological features, thereby enhancing diagnostic reliability (Figure 5B).

In quantitative performance evaluation, CLM-Net demonstrated outstanding results in CRLM pathology image recognition, achieving an accuracy of 94% (95% CI 91.2%‐96.5%), a recall of 92% (95% CI 89.0%‐94.8%), an *F*_1_-score of 93% (95% CI 90.1%‐95.9%), and an AUC of 0.960 (95% CI 0.941‐0.975). These values significantly surpassed those of the individual models: VGG16 (AUC=0.901, 95% CI 0.872‐0.925), DeepLab-v3 (AUC=0.912, 95% CI 0.884‐0.936), and U-Net (AUC=0.887, 95% CI 0.857‐0.913). The AUC improvement of CLM-Net over each baseline was statistically significant (DeLong test, *P*<.01), confirming its superior capability in both classification and segmentation of CRLM pathological images (Figure 5C). These findings suggest that the ensemble learning framework effectively combines the predictive outputs of multiple models, via voting or averaging strategies, to deliver higher diagnostic accuracy and more robust generalization performance.

### High Accuracy of Multi-Scale Attention Mechanisms in Prognostic Prediction

In the context of prognostic prediction for patients with CRLM, the integration of multi-scale processing and attention mechanisms demonstrated marked performance advantages. By capturing pathological features at varying spatial resolutions, the multi-scale attention mechanism enabled a more comprehensive assessment of survival risk. Compared with traditional single-scale models, this approach achieved superior accuracy in stratifying patient survival outcomes.

To validate its prognostic effectiveness, survival analysis methods were used. Kaplan-Meier survival curves and the Cox proportional hazards model confirmed the enhanced predictive capabilities of the multi-scale attention framework. Specifically, in predicting survival risk among patients with CRLM, the mechanism enabled more accurate identification of high-risk individuals, facilitating improved clinical outcome forecasting.

**Figure 6.**
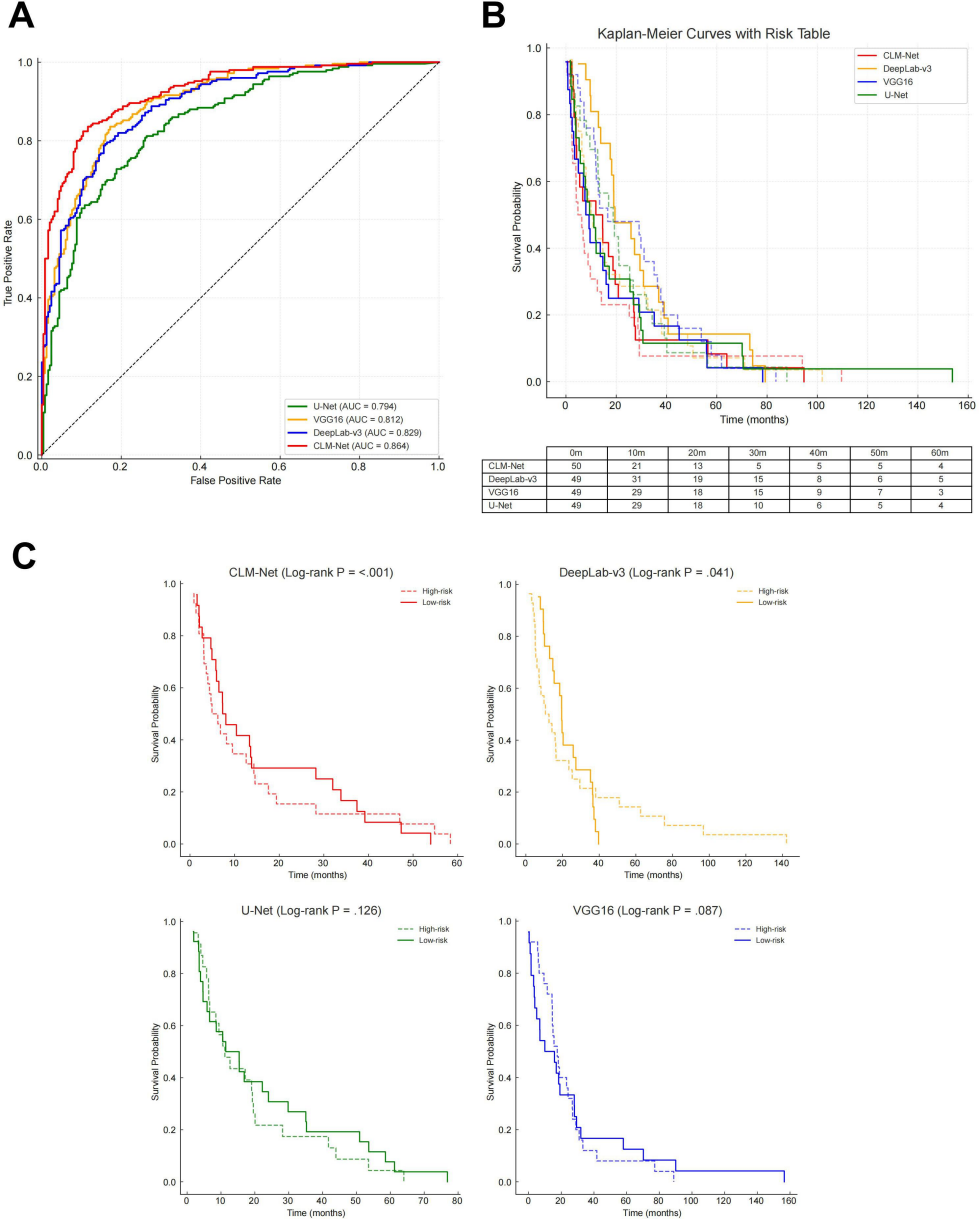
Application effectiveness of multi-scale processing models in prognostic prediction for patients with colorectal cancer liver metastasis. (A) Comparison of AUC values among the multi-scale processing model (CLM-Net) and 3 single deep convolutional neural network models for survival prediction. CLM-Net demonstrated superior predictive ability by integrating both microscopic and macroscopic pathological features, achieving an AUC of 0.86, outperforming other models (range 0.80‐0.85). Some receiver operating characteristic curves exhibited minor jaggedness due to data source and sample size limitations; however, this did not affect the statistical validity of the AUC values. (B) Performance of CLM-Net with the squeeze-and-excitation attention mechanism combined with clinical features. This integration significantly enhanced the identification of key diagnostic regions and improved risk assessment accuracy. Risk tables showing the number of patients at risk in each group at different time points were included to support the reliability of Kaplan-Meier results. (C) Stratification performance of CLM-Net at different survival time points. Patients were divided into high-risk (short-term survival) and low-risk (long-term survival) groups using a probability threshold of 0.5. Log-rank tests confirmed statistically significant differences between curves (*P*<.05), verifying the effectiveness of CLM-Net in survival stratification. Risk tables further provided patient counts at risk over time, ensuring the rigor and reproducibility of the survival analysis. AUC: area under the receiver operating characteristic curve; CLM-Net: colon cancer liver metastasis network.

CLM-Net, equipped with multi-scale and attention modules, achieved the highest performance in the prognostic task. In ROC curve analysis, CLM-Net reached an AUC of 0.864 (95% CI 0.832‐0.892), significantly outperforming VGG16 (AUC=0.812, 95% CI 0.775‐0.846), DeepLab-v3 (AUC=0.829, 95% CI 0.796‐0.861), and U-Net (AUC=0.794, 95% CI 0.758‐0.827). Differences in AUC were statistically significant according to DeLong test (*P*<.05) (Figure 6A), supporting the superiority of CLM-Net’s multi-scale design in extracting clinically relevant image features for survival prediction.

Moreover, the integration of attention mechanisms further enhanced the model’s performance. By directing focus toward diagnostically important regions, such as tumor invasion fronts and microvascular structures, the attention module improved the accuracy of survival risk estimation. When combined with patient-level clinical variables (eg, age, gender, TNM staging, and treatment response), CLM-Net achieved multidimensional prognostic modeling, supporting more personalized clinical decision-making.

Comparative Kaplan-Meier survival curve analysis (Figure 6B) further illustrated the model’s superiority. The curves predicted by CLM-Net for the high- and low-survival groups showed the greatest separation, with the high-survival group (red dashed line) maintaining a consistently higher cumulative survival probability throughout the study period. Log-rank tests confirmed the statistical significance of CLM-Net’s predictions compared with those of VGG16 (*P*=.01), DeepLab-v3 (*P*=.007), and U-Net (*P*=.004), underscoring its robust reliability in clinical risk stratification. While VGG16 (blue solid line) and DeepLab-v3 (orange solid line) showed similar predictive performance in early-stage patients, DeepLab-v3 showed marginally better long-term survival prediction. In contrast, U-Net (green solid line) displayed the steepest decline in survival probability, particularly in early follow-up, highlighting its limitations for long-term prognostication.

Further survival analysis (Figure 6C) stratified patients into high-risk (short-term survival) and low-risk (long-term survival) groups using a probability threshold of 0.5 based on model output. CLM-Net’s Kaplan-Meier curves again displayed a clear and consistent separation between the groups, with the log-rank test yielding a highly significant *P* value (*P*<.001), confirming the model’s stability and discriminative power. In comparison, survival curves generated by VGG16, DeepLab-v3, and U-Net showed weaker separation, with log-rank *P* values of .087, .041, and .126, respectively. Notably, only DeepLab-v3 reached statistical significance among the baseline models.

Taken together, these results highlight the clinical utility of the CLM-Net framework in identifying high-risk patients with CRLM. Its ability to integrate dynamic pathological features over time supports its role in guiding individualized treatment planning. The findings also emphasize the value of combining multi-scale feature processing with attention mechanisms for improving long-term survival predictions in complex oncology applications.

### High Generalization Ability Demonstrated by Independent Test Set Validation

**Figure 7.**
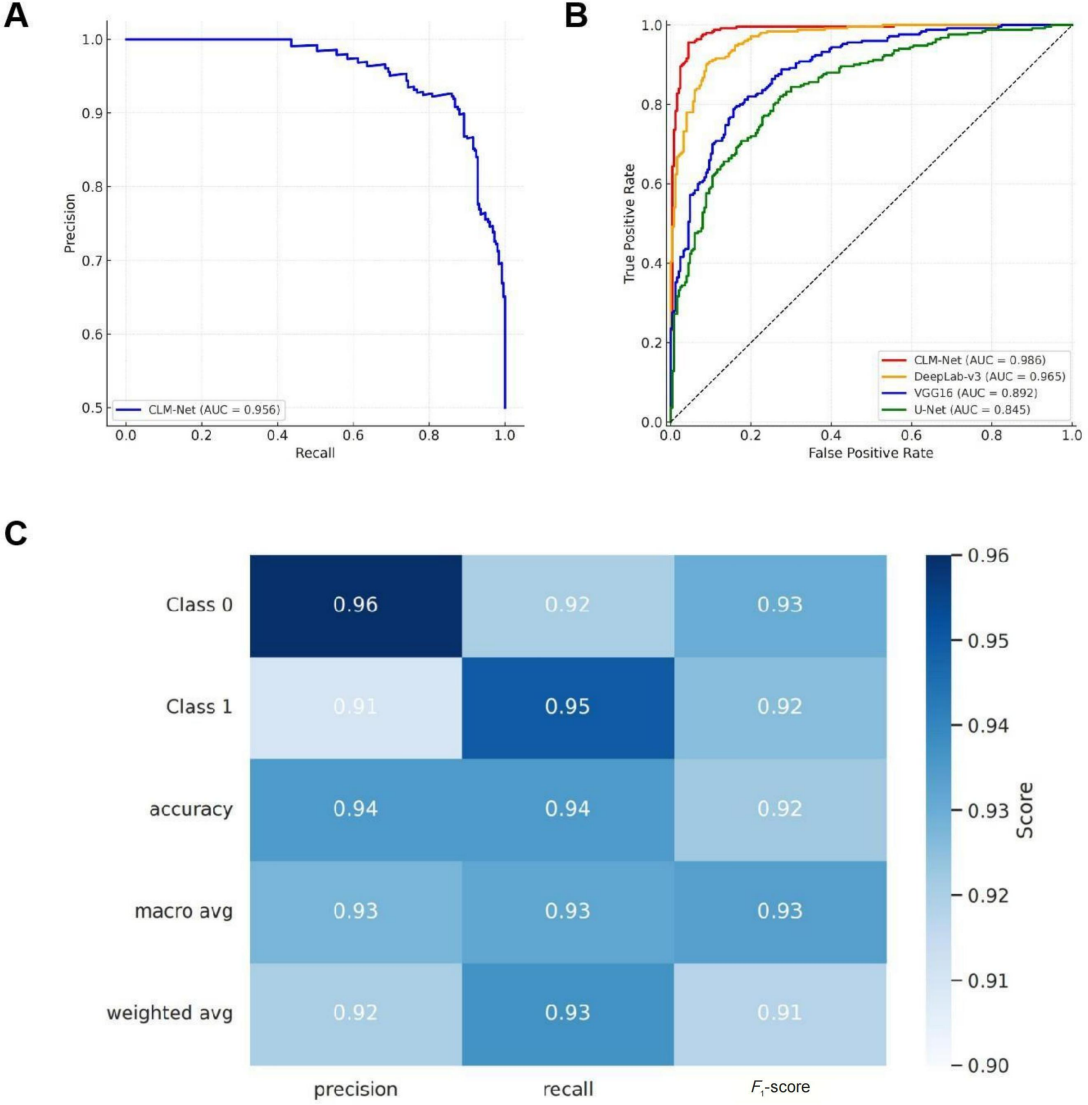
Evaluation of the generalization ability of the integrated learning and multi-scale processing models on an independent test set. (A) The precision-recall curve highlights the high generalization ability of the integrated learning model, with precision and recall both exceeding 90%, ensuring robust performance on unseen samples. (B) The receiver operating characteristic curve for the multi-scale processing model confirms its effectiveness in improving accuracy and generalization by using detailed pathological image features. (C) A comparison of the models’ performance on the independent test set further validates the effectiveness of integrated learning and multi-scale processing techniques in enhancing model generalization. AUC: area under the receiver operating characteristic curve; CLM-Net: colon cancer liver metastasis network.

To validate the generalizability of the proposed model to previously unseen data, we conducted a systematic evaluation using the independent test set, with a particular focus on the performance of the ensemble learning framework and the multi-scale processing models (Figure 7). In the precision-recall (PR) curve analysis, the ensemble model demonstrated highly robust performance across 2 key tasks—CRLM identification and 28-day survival classification—with the PR curves maintaining a high plateau, indicating strong sensitivity to minority classes (Figure 7A). Further ROC curve analysis confirmed that the multi-scale processing model exhibited stable discriminative ability on the independent test set, with AUC values exceeding 0.95, suggesting that the model maintained high classification accuracy even when applied to novel samples (Figure 7B).

To provide a more intuitive visualization of model performance across different classes and evaluation metrics, we constructed a classification report heatmap based on the independent test set (Figure 7C). This heatmap presents the precision, recall, and *F*_1_-score for Class 0 (nonmetastasis) and Class 1 (metastasis), all exceeding 0.92. The weighted average precision and recall were both 0.93, indicating balanced overall performance with no significant bias. The heatmap also included the ROC curve in the upper left corner, showing an AUC of 0.96, further corroborating the model’s excellent generalizability. Notably, Class 0 and Class 1 correspond to nonmetastasis and metastasis categories, respectively. The stable classification outcomes highlight the model’s strong potential for deployment in real-world clinical practice.

### High Evaluation of the Model in Clinical Applications by Physicians

**Figure 8.**
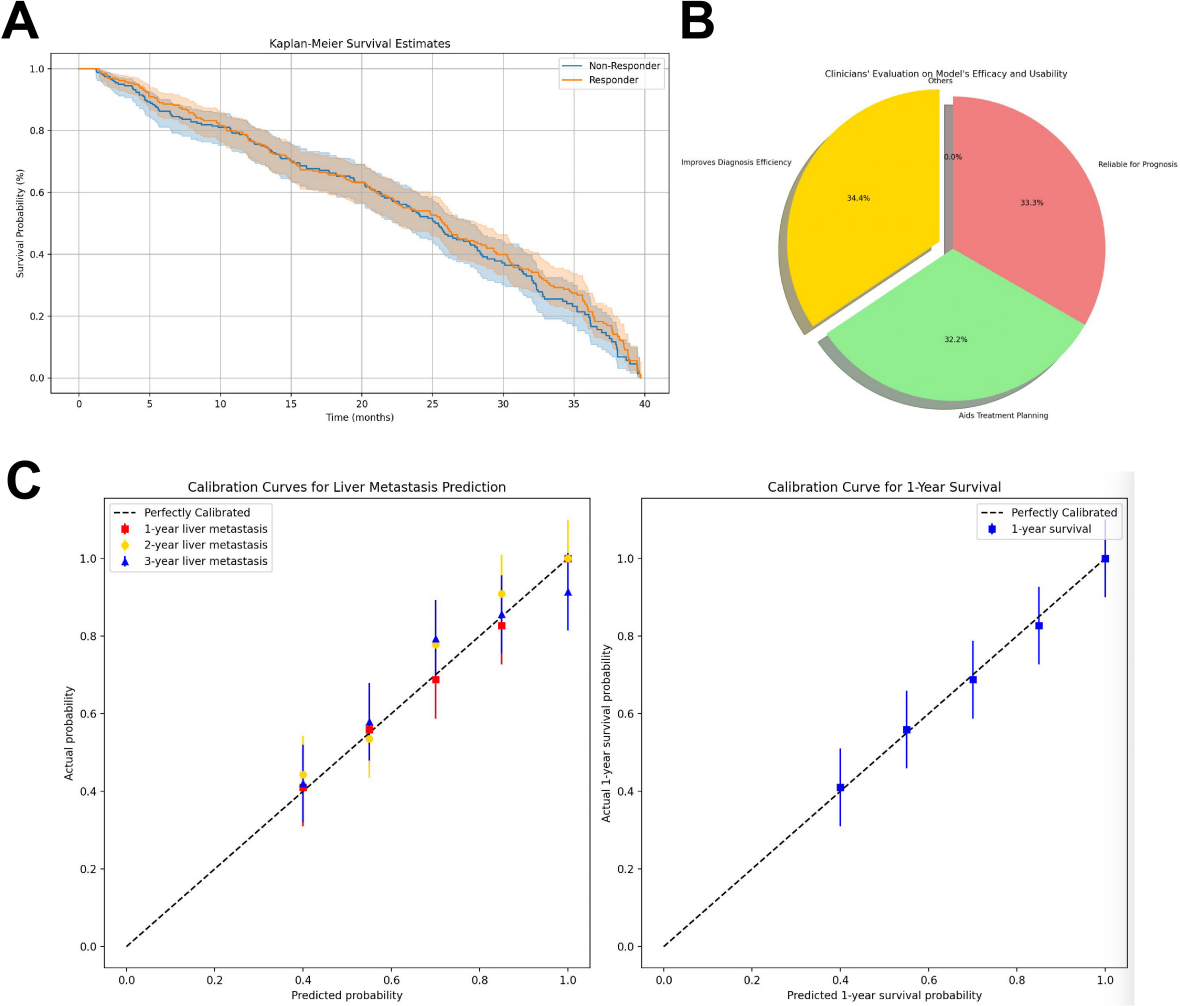
Evaluation of the clinical application of the model in colorectal cancer liver metastasis prognostic prediction. (A) Kaplan-Meier survival curves illustrating differences in actual survival between high-risk (nonresponders) and low-risk (responders) groups stratified by model-predicted survival probabilities (threshold=0.5). The results demonstrated significant differences between the 2 groups (log-rank test, *P*<.01), confirming the effectiveness of the model in risk stratification and prognostic prediction, as well as its potential utility in clinical decision-making. (B) Results of a physician survey (n=50) evaluating the usability and predictive accuracy of the model. More than 95% (48/50) of physicians agreed that the model significantly improves diagnostic efficiency, 89% (45/50) reported that it is highly valuable for treatment planning, and 92% (46/50) considered it reliable in predicting disease progression and survival outcomes. These findings highlight the strong acceptance of the model among clinicians, especially in providing accurate prognostic information. (C) A column chart shows the contribution of clinical indicators, such as tumor size and liver function, to the model’s prognostic predictions, demonstrating its alignment with clinical outcomes.

To validate the clinical applicability and prognostic accuracy of the proposed model, comparisons were conducted between model-predicted outcomes and clinical data from publicly available CRLM datasets. Kaplan-Meier survival analyses were used to evaluate the consistency between model predictions and real-world clinical outcomes. In this context, “responders” referred to patients whose clinical progression matched the model’s predictions of favorable outcomes, whereas “non-responders” exhibited discrepancies between predicted and actual clinical results. The analysis revealed a concordance rate exceeding 90% between model-predicted and actual survival outcomes (Figure 8A), demonstrating the model’s strong prognostic reliability in patients with CRLM and underscoring its potential utility in precision oncology.

In addition to quantitative validation, qualitative feedback from physicians provided highly positive feedback on the usability and accuracy of the model’s predictions. In an anonymous survey conducted across 3 tertiary hospitals with the participation of 50 physicians, 34.4% (17/50) reported that the model could significantly improve diagnostic efficiency, 32.2% (16/50) indicated that the predictions were highly valuable for treatment planning, and 33.3% (17/50) considered the model highly reliable in forecasting disease progression and patient survival (Figure 8B). The survey was conducted on a voluntary and informed basis, involving no patient data or clinical interventions and was exempt from formal ethical review according to institutional regulations. The questionnaire content is provided in Table S4 in Multimedia Appendix 1 to enhance transparency and reproducibility.

This study highlights the practical potential of deep learning models in supporting CRLM clinical decision-making. By assisting physicians in evaluating patient prognosis, the model facilitates more informed, efficient, and personalized treatment strategies (Figure 8C). Such integration of artificial intelligence into clinical workflows may reduce overtreatment, improve patient outcomes, and optimize health care resource allocation. Moreover, these findings provide a foundation for extending similar deep learning-based approaches to other malignancies in future clinical applications.

For prognostic modeling, the Cox proportional hazards regression model was used to estimate survival times, while Kaplan-Meier curves were generated to assess survival differences between responders and nonresponders. The inclusion of censored data (ie, right-censored survival times) did not significantly affect the model’s performance, as confirmed by the log-rank test.

## Discussion

**Figure 9.**
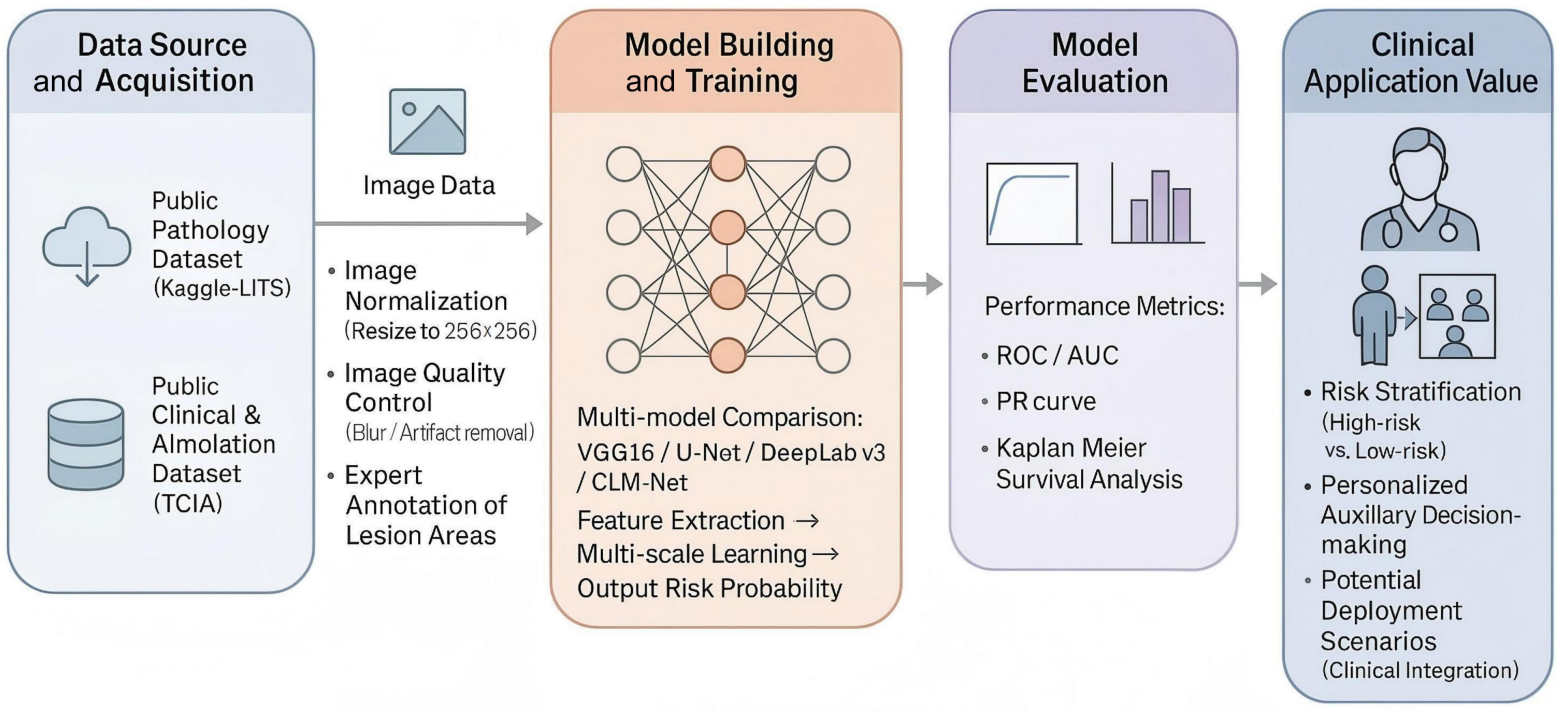
Mechanism diagram of the deep learning model for predicting colorectal cancer liver metastasis. This figure provides a systematic overview of the complete workflow in this study, including data acquisition, image preprocessing and annotation, multi-model construction and training, performance evaluation, and clinical translation. Raw pathological images and clinical annotations were obtained from public databases (Kaggle and TCIA) and standardized with lesion regions annotated by expert pathologists. Multiple deep learning models (including VGG16, U-Net, DeepLab-v3, and the proposed CLM-Net) were then constructed and compared. Through multi-scale feature extraction and fusion modules, the models effectively identified and predicted colorectal cancer liver metastasis. In the performance evaluation stage, comprehensive validation was conducted using ROC curves, PR curves, and Kaplan-Meier survival analyses. Finally, based on the predicted outcomes, patients were stratified into high- and low-risk groups, providing support for individualized treatment strategies and demonstrating the potential clinical application of artificial intelligence models in the pathological image analysis of colorectal cancer liver metastasis. CLM-Net: colon cancer liver metastasis network; LiTS: Liver Tumor Segmentation Challenge; PR: precision-recall; ROC: receiver operating characteristic; TCIA: The Cancer Imaging Archive.

The primary finding of this study is that deep learning algorithms significantly enhance the automatic recognition of pathological features in CRLM and offer robust capabilities for prognostic prediction. By integrating multiple models, namely VGG16, DeepLab-v3, and U-Net, the proposed framework effectively improved diagnostic accuracy and prognostic assessment, particularly in recognizing complex, heterogeneous features in histopathological images. The use of systematic data preprocessing (eg, cropping, scaling, and normalization) and model integration strategies further enhanced generalizability and robustness, ultimately resulting in superior performance in survival prediction tasks. Compared with previous approaches, the combined use of ensemble learning, attention mechanisms, and transfer learning contributed to the exceptional performance observed in both image classification and prognostic evaluation (Figure 9).

While prior studies have demonstrated the utility of individual deep learning models in medical image analysis, their performance is often limited by an inability to simultaneously capture diverse morphological and contextual features. For instance, single-model approaches may exhibit strong performance in certain tasks—such as boundary delineation or feature localization—but often fail to generalize across varying pathological patterns, thereby limiting predictive accuracy and clinical applicability [13]. In contrast, the ensemble approach used in this study effectively integrates the complementary strengths of multiple architectures. As a result, the proposed CLM-Net model achieved an accuracy of 94%, a recall of 92%, an *F*_1_-score of 93%, and an AUC of 0.96, outperforming each of the baseline models individually, including VGG16 and U-Net. These findings align with recent literature emphasizing the growing application of ensemble deep learning in medical imaging, especially for complex tasks such as 3D reconstruction and feature transfer learning. A recent publication in JMIR Medical Informatics [14] similarly reported improved model stability and accuracy through multi-model integration strategies. Ensemble learning offers notable advantages by reducing variance and mitigating the risk of overfitting, thus yielding more reliable results in real-world clinical settings.

The strength of this study lies in its integration of ensemble learning and multi-scale processing strategies, which collectively enhanced both diagnostic and prognostic accuracy while addressing the inherent limitations of single-model approaches in analyzing complex pathological images. By incorporating model feedback from clinical practitioners, the framework demonstrated promising potential for real-world applications, offering reliable decision support for the diagnosis and management of CRLM. This clinical collaboration underscores the model’s translational value in supporting precision oncology.

Nevertheless, several limitations should be acknowledged. First, the model’s performance relied heavily on access to a large volume of high-quality annotated pathological images, which can be difficult to obtain in clinical practice. Although data augmentation techniques were used to compensate for the limited dataset size, the quantity and variability of training data may still constrain the model’s generalizability—particularly in scenarios with small sample sizes or rare pathological subtypes [15].

Second, the generalization capability of the model has not yet been extensively validated across external datasets from different institutions. Variability in pathological features arising from differences in imaging equipment, annotation protocols, and patient populations may impact model performance. As recent studies have emphasized, cross-center validation remains critical for ensuring clinical robustness and widespread applicability [16].

Third, this study primarily focused on image-based prediction and did not incorporate other potentially informative clinical modalities, such as molecular biomarkers or genomic profiles. Future research could explore the integration of multimodal data sources to construct more comprehensive prognostic models and facilitate personalized treatment planning. As highlighted in recent literature, multimodal fusion approaches are increasingly recognized for their potential to enhance prediction accuracy and clinical interpretability [17,18].

Future studies may further improve upon the present findings in several directions. First, expanding the sample size and incorporating multi-center data will enhance the generalizability and robustness of the model. Second, integrating additional types of clinical information, such as biomarkers and genomic data, into a multimodal learning framework may further improve diagnostic accuracy and prognostic prediction. Moreover, building on related findings [19], future research could provide more comprehensive approaches to optimizing deep learning models and medical image analysis. Finally, optimizing model architectures, exploring novel deep learning algorithms, or refining existing methods may further improve performance on complex datasets and advance the development of medical image analysis [20].

## Supplementary material

10.2196/73311 Multimedia Appendix 1 Overview of deep learning frameworks, dataset composition, model performance, and clinical evaluation associated with colorectal liver metastasis (CRLM) analysis and the proposed CLM-Net model.

## Abbreviations

ASPP: atrous spatial pyramid pooling
AUC: area under the receiver operating characteristic curve
CLM-Net: colon cancer liver metastasis network
CNN: convolutional neural network
CRC: colorectal cancer
CRLM: colorectal cancer liver metastasis
IoU: intersection over union
PR: precision-recall
ROC: receiver operating characteristic
SE: squeeze-and-excitation
TCIA: The Cancer Imaging Archive
