# Supplementary material for: Automatic Recognition and Prognostic Prediction of Colorectal Liver Metastases Using a Multi-Scale Deep Learning Framework: Model Development and Validation Study
Source: JMIR Med Inform. 2026 Apr 7;14:e73311. doi: 10.2196/73311 (PMC13055949; doi:10.2196/73311)
Supplement: Multimedia Appendix 1 [file medinform-v14-e73311-s001.docx]

**Table S1. Summary of Representative Deep Learning Frameworks Applied to Colorectal Liver Metastasis (CRLM) Analysis**

| No. | Study & Year | Model / Framework | Application Task | Data Source | Advantages | Limitations |
| --- | --- | --- | --- | --- | --- | --- |
| 1 | Li et al., 2024 (arXiv) | Bi-LSTM + Multi-plane structure | CRLM prediction based on CECT sequence | In-house 5D-CT sequence (n=269) | Captures temporal dynamics; suitable for multiphase data | Highly dependent on temporal data; room for AUC improvement |
| 2 | Anderson et al., 2022 (Hybrid-WNet) | 3D Hybrid-WNet | Liver metastasis segmentation on CT | 3D-IRCADb + LiTS datasets | Fast segmentation; good clinical scoring | Limited sensitivity to small lesions |
| 3 | Guo et al., 2024 (Eur J Surg Oncol) | Random Forest (RF) and ML models | Risk prediction based on clinical features | SEER database + external validation | High AUC; interpretable; large-scale data | No image-level analysis |
| 4 | Huang et al., 2025 (4D-ACFNet, arXiv) | 4D Spatiotemporal Attention Model | Dynamic prognosis prediction | 197 CRLM multimodal cases | Innovative 4D architecture with multimodal fusion | High computational cost; not yet peer-reviewed |
| 5 | Zhang et al., 2023 (PMC) | ResNet-50 + Cox Regression | CRLM prediction using digital pathology | 611 CRC pathology cases (multi-center) | Pathology-based prediction without imaging | No segmentation; only risk scoring |
| 6 | This study (CLM-Net) | VGG16 + DeepLab-v3 + U-Net + SE + CRF + LR/RF | Histopathological classification + survival prediction | Kaggle + TCIA (n=197) | Integrated multi-model architecture; excellent performance in both classification and prognosis | Complex implementation; requires external validation and training resources |

**Table S1 References**

1. Li X, Xiao H, Weng W, et al. MPBD-LSTM: A predictive model for colorectal liver metastases using time series multi-phase contrast-enhanced CT scans.In: Medical Image Computing and Computer-Assisted Intervention – MICCAI 2023. LNCS 14225; 2023. p. 379–388.

2. Anderson BM, Rigaud B, Lin YM, et al. Automated segmentation of colorectal liver metastasis and liver ablation on contrast-enhanced CT images. *Front Oncol*. 2022;12. doi:10.3389/fonc.2022.886517

3. Guo Z, Zhang Z, Liu L, et al. Machine learning for predicting liver and/or lung metastasis in colorectal cancer: A retrospective study based on the SEER database. *European Journal of Surgical Oncology*. 2024;50(7):108362. doi:10.1016/j.ejso.2024.108362

4. Li Z, Yang W, Su Y, et al. 4D-ACFNet: A 4D Attention Mechanism-Based Prognostic Framework for Colorectal Cancer Liver Metastasis Integrating Multimodal Spatiotemporal Features. *arXiv.org*. Published online March 12, 2025. doi:10.48550/arXiv.2503.09652

5. Xiao C, Zhou M, Yang X, et al. Accurate Prediction of Metachronous Liver Metastasis in Stage I-III Colorectal Cancer Patients Using Deep Learning With Digital Pathological Images. *Front Oncol*. 2022;12. doi:10.3389/fonc.2022.844067

**Table S2. Distribution of Patient Classes Across Training, Validation, and Independent Test Sets**

| Dataset Split | Non-metastasis (Class 0) | Metastasis (Class 1) | Total |
| --- | --- | --- | --- |
| Training Set (70%) | 348 | 352 | 700 |
| Validation Set (15%) | 76 | 74 | 150 |
| Test Set (15%) | 73 | 77 | 150 |
| Total | 500 | 500 | 1000 |

**Table S3. Comparative Performance of CLM-Net and Baseline Deep Learning Models in Prognostic Prediction**

| Model | Optimized Learning Rate | Accuracy (%) | Recall (%) | F1 Score (%) | AUC |
| --- | --- | --- | --- | --- | --- |
| VGG16 | 1e-4 | 89.2 | 87.5 | 88.3 | 0.91 |
| ResNet50 | 5e-5 | 90.1 | 88.3 | 89 | 0.925 |
| DenseNet121 | 1e-5 | 91.4 | 90 | 90.7 | 0.938 |
| InceptionV3 | 3e-5 | 90.8 | 89.1 | 89.6 | 0.932 |
| CLM-Net (Ours) | 1e-4 | 94 | 92 | 93 | 0.96 |

**Table S4. Questionnaire Survey Results from Clinicians on the Usability and Clinical Utility of CLM-Net**

| Question ID | Question (English) | Options (English) |
| --- | --- | --- |
| Q1 | 1. How do you evaluate the clinical utility of this model? | Very high; High; Moderate; Low; Very low |
| Q2 | 2. Do you think the model can improve your diagnostic efficiency? | Yes; No; Not sure |
| Q3 | 3. Is the model's prediction helpful in treatment planning? | Yes; No; Not sure |
| Q4 | 4. How would you rate the model's accuracy in disease progression prediction? | Very accurate; Relatively accurate; Moderate; Inaccurate |
| Q5 | 5. Would you be willing to use this model in your future clinical practice? | Yes; No; Depends |
| Q6 | 6. Is the model interface and operation user-friendly? | Very friendly; Relatively friendly; Moderate; Not friendly |
| Q7 | 7. Do you think the model has potential for further clinical promotion? | Fully; Somewhat; Not sure; No potential |
| Q8 | 8. Are you concerned that model misjudgment may affect clinical decisions? | Yes; No; Not sure |
| Q9 | 9. In which aspects do you hope the model can be further improved? (Multiple choice) | Accuracy; Speed; UI friendliness; Compatibility with workflow; Others |
| Q10 | 10. Your professional title/role: | Attending physician; Associate chief physician; Chief physician; Researcher; Others |
